# The association between maternal parenting perceived by early childhood teachers and burnout: the mediating effect of self-compassion and teacher efficacy

**DOI:** 10.3389/fpsyg.2023.1229065

**Published:** 2024-01-30

**Authors:** Yujin Jang, Yea-Ji Hong

**Affiliations:** ^1^Department of Early Childhood Education, Gachon University, Seongnam-si, Republic of Korea; ^2^Department of Child Studies, Inha University, Incheon, Republic of Korea

**Keywords:** burnout, early childhood teacher, maternal parenting, self-compassion, teacher efficacy

## Abstract

Given the increasing importance of early childhood teachers, this study aims to identify whether past maternal parenting is related to burnout through the double mediation of teacher self-compassion and teacher efficacy, using model comparison. To this end, a survey was conducted with 329 early childhood teachers in Korea. The positive maternal parenting perceived by teachers was negatively related to burnout through teachers’ self-compassion and efficacy. Positive maternal parenting reduced burnout by increasing self-compassion and consequently teacher efficacy. Teachers’ self-compassion was more closely related to burnout than teacher efficacy, which was treated as an important variable in relation to teachers. These results suggest that early childhood teachers’ self-compassion is a significant variable in relation to burnout and suggestions are provided for specific support programs to promote teacher self-compassion.

## 1 Introduction

In Korea, 81.1% of infants and 91.3% of two-year-olds are currently receiving childcare at daycare centers (Korea Ministry of Health and Welfare, 2020). Early childhood teachers in Korea experience burnout due to physical and mental fatigue, low social awareness, poor wages and welfare benefits, and the daily care of infants and children until late. When examining the proportion of people experiencing high stress in their jobs, the proportion working in education was found to be the highest compared with other occupational fields (Wettstein et al., 2021). Burnout is a physical and mental depletion phenomenon that occurs in humans due to excessive stress, and rather than appearing suddenly one day, it occurs as a result of continuous and repetitive stress in a situation where people are in a close relationship for a long time. In the workplace context, burnout is a prolonged response to chronic emotional and interpersonal stressors on the job and is constructed from three factors: emotional exhaustion, a feeling of cynicism, and a sense of inefficiency (Maslach et al., 2001). It is known to be among the variables with the greatest adverse effect on teachers’ social and emotional abilities. Teachers who have experienced psychological burnout cannot provide an appropriate environment for infants and children, experience emotional difficulties, and treat children negatively and coldly. In previous studies, teacher burnout was not only detrimental to teachers but also associated with low-quality teaching and interactions, ultimately leading to negative childhood development (Greenberg et al., 2016; Sandilos et al., 2020). Given that children spend more time in educational institutions, and the social responsibility for parenting has increased, teacher burnout should be urgently addressed. Therefore, it is necessary to identify the various variables related to teacher burnout.

Parenting affects the entire life, affecting human psychosocial development and health not only in childhood but also in adolescence and adulthood. Parents play a critical role in their children’s social–emotional development and adjustment (Eisenberg et al., 1998; Bornstein et al., 2018; Spinrad et al., 2020). This study examines the effects of past maternal behavior as perceived by early childhood teachers. Many previous studies on intergenerational transfer between adults and parents have focused on the transfer of attachment. This is presumably because attachment results in parenting characteristics; adults must recall at least 10–30 years prior in order to evoke past parenting. However, this study considers the impact of past maternal parenting on teachers’ psychosocial outcomes for two reasons. First, as the age of infants and children entering educational institutions has become younger, and the time spent at educational institutions has increased, interest in “teacher parenting” has grown, emphasizing the role of teachers as educators. There is an argument that humans are raised by their parents, and many people do not have multiple parents. Therefore, it is difficult to experience various parenting styles, and we acquire the ways in which our parents raised us. Ultimately, teachers are likely to raise infants and children as they have been raised. Second, when comparing the measured items, the parenting concept can provide more practical and specific behavioral guidelines than attachment. This is because by identifying the four parenting sub-factors of past affection, rejection, autonomy, and control as perceived by teachers, various dimensions of analysis are possible and practical suggestions for parenting behavior can be obtained. In this study, we investigate whether maternal parenting, as perceived by teachers, affects the psychosocial characteristics of current early childhood teachers.

The intergenerational transfer of parenting can be explained using social learning theory and modeling (Bandura, 1997). Social learning theory explains how people learn behaviors, thinking, emotions, values, and attitudes by observing others. In modeling, an individual selects someone as a role model and applies their behavior to him/herself. Individuals reproduce that behavior after observing the model’s specific behavior and its consequences. Children are more likely to model their parents without special reinforcement. In addition, family system theory suggests that children develop not only within the context of dual relationships with parents but also within extended family networks. Previous studies on grandparents’ parenting have demonstrated the intergenerational transmission of harsh and aggressive parenting (Capaldi et al., 2008), confirming that the parenting of grandparents affects the social competence of children through the parenting of parents (Jang, 2020). In another study, grandmothers’ parenting was significantly related to children’s internalization problems, and this relationship was mediated by the parenting of perceived parents (Li et al., 2018). While, the teacher-child relationship is not a parent–child relationship, it is possible that the teacher’s attitude toward parenting is influenced by his or her parents. Past parenting experiences of early childhood teachers are likely to affect the way they treat small, vulnerable children when they are raised and educated.

Previous studies have shown that burnout is affected by the people around you. When supported by significant others, such as family or friends, burnout is low and social support is an important variable that reduces burnout (Jacobs and Dodd, 2003). Excessively controlling parenting is known to promote burnout in children (Shin et al., 2012). If parents do not recognize their children’s autonomy and control, and protect them excessively, the children do not have the opportunity to make decisions on their own. Lacking this experience is likely to have a negative effect on subsequent behaviors by lowering self-efficacy. In fact, helicopter parenting, characterized by excessive control and the suppression of autonomy, may hinder the development of self-control skills among college students, which is associated with feelings of burnout (Love et al., 2020). Contrastingly, a supportive parenting environment that is positive and promotes autonomy was found to prevent burnout in adolescents (Álvarez et al., 2019). Therefore, parenting, as perceived by teachers, is related to teacher burnout.

Among the main variables affecting early childhood teacher performance is teacher efficacy. Teacher efficacy is derived from self-efficacy, which refers to the belief in one’s ability to organize and execute the course of action necessary to achieve a specific goal (Bandura, 1997). More than any other variable, self-efficacy is the most efficient concept for predicting human achievement and motivation (Jang and Lee, 2021). Regardless of how much knowledge and skills humans have, they do not act without a positive belief in their abilities (Bandura, 1997). Teacher efficacy is a self-assessment of teacher competence that has a strong influence on teacher behavior (Tschannen-Moran and Hoy, 2001). Teachers with high self-efficacy are sensitive to their children’s needs, teach passionately, and have high levels of job satisfaction (Wolters and Daugherty, 2007; Skaalvik and Skaalvik, 2014). Bandura (1997) noted that self-efficacy is primarily affected by the family, especially parents, and that general efficacy can later be transferred to teacher efficacy with specificity. Early childhood teachers should be able to manage themselves in a free and active environment, performing various education-related tasks. However, children who are raised negatively by their parents may have trouble solving their own difficulties due to their low self-efficacy, even as adults, and may not be able to cope properly with the various stressors of the educational field. An accumulation of such stress can lead to job burnout.

Many previous studies have argued that there is an association between teacher efficacy and burnout. Since efficacy is a useful concept for predicting independent behavior and achievement, it is an easily predictable result. In a study of Chinese teachers, teachers’ self-concept influenced burnout through teacher efficacy, which has been suggested as a model of the cyclical nature of teacher efficacy (Zhu et al., 2018). In a meta-analysis of studies conducted in various Western countries, teacher efficacy in classroom management showed a significant negative correlation with all three aspects of burnout (Aloe et al., 2014). In addition, numerous studies on the association between teacher efficacy and burnout reported a negative correlation between teacher efficacy and burnout.

Recently, as the need to acknowledge and care for one’s self warmly, as a human being has been raised, the concept of “self-compassion” is drawing attention in the psychological community. Self-compassion is a concept derived from Buddhist psychology, based on kindness, which sympathizes with other people’s pain and recognizes it without avoiding it. Here, compassion is a combination of a heart of infinite love and a heart of regret, meaning that you love and pity someone endlessly. Ultimately, self-compassion means mercy toward oneself; therefore, self-compassion can be defined as humans understanding their pain as a universal experience, treating themselves kindly with a mind-taking approach, and fully experiencing the object of pain (Neff, 2003b). Neff (2009) emphasizes that recognizing one’s thoughts and feelings is a prerequisite for self-compassion. Self-compassion comprises three elements: self-kindness, common humanity, and mindfulness. Self-kindness refers to being kind to yourself as you would with others, which refers to being tolerant of all parts of yourself, even if your thoughts, actions, and feelings seem inappropriate. Common humanity refers to the attitude in which all humans acknowledge their imperfections and view pain as something that anyone living life can experience. Finally, mindfulness refers to the non-judgmental observation of painful thoughts and emotions without their suppression or exaggeration (Neff, 2003b). People who practice mindfulness try to stay in the “here and now” when they feel pain, carefully observing their current experience and fully experiencing the pain (Neff, 2003b; Germer, 2009). Ultimately, when these three factors are equally satisfied, humans can have a benevolent attitude toward themselves.

Although self-compassion has recently attracted attention, studies on parenting and self-compassion are relatively limited (Cohen and Naaman, 2022). A few studies have suggested that warm and responsive parenting are associated with children’s self-compassion (Dakers and Guse, 2022), and positive parenting is associated with higher self-compassion and lower depression in both British and Chinese adolescents (Zhao et al., 2021). The argument that self-compassion can be cultivated through practice and training, further raises interest in the concept. Moreover, it is worth paying attention to situations where teachers’ mental health is threatened and self-compassion functions as a protective factor that contributes to human resilience after exposure to negative life events (Neff, 2009; Neff and McGehee, 2010). Certainly, one cannot change their past parenting, and it is difficult to overcome psychological difficulties alone. Thus, if self-compassion mediates teachers’ perceived parenting and psychosocial health, and if teachers are provided with specific training to increase self-esteem, it could help early childhood teachers.

For a positive interaction with infants and children, teachers should manage and express their emotions appropriately, and express appropriate emotions “as a teacher” apart from their own emotions. Teachers are in a context where they must manage and sometimes suppress their emotions, not only in their relationships with children but also with parents; thus, their self-compassion can be a factor in protecting their mental health. In a study of nurses in Korea, strengthening self-compassion helped prevent burnout (Jang et al., 2022); and another study of college students in the United States also found that self-compassion mediated the relationship between narcissism and burnout (Barnett and Flores, 2016). However, few if any studies on self-compassion in early childhood teachers exist, and none were found that consider teacher efficacy and burnout, which are important variables for early childhood teachers. Thus, this study seeks to help understand the psychological state and self-compassion of early childhood teachers.

While previous studies on self-compassion and teacher efficacy are difficult to find, this study assumes that self-compassion affects teacher efficacy. This assumption is theoretically based on Bandura’s (1997) physiological and affective states, and that people rely on physical information transmitted by physiological and emotional states to judge their abilities. Bandura (1997) argued that human efficacy is influenced by four factors: experience of mastery, vicarious experience, verbal persuasion, and physiological and emotional states. Among these sources of information, experience of mastery has the greatest influence on self-efficacy. However, people tend to expect more success when there is no awakening than when they are emotionally tense and restless. In other words, human efficacy can be enhanced by improving physical conditions, reducing stress levels and negative emotional tendencies, and correcting misinterpretations of the physical condition (Bandura, 1991). Considering that teacher efficacy is a belief in one’s ability as a teacher and is one of the various human efficacies, it is also likely to be affected through physiological and emotional states. Previous studies have argued that teachers should treat themselves kindly, and if this process is not conducted properly, they are likely to devalue their self-efficacy and achievement (Akpan and Saunders, 2017), suggesting a relationship between self-compassion and teacher efficacy. A self-compassionate teacher will love herself, be generous, accept herself kindly and feel comfortable. In recent studies, higher levels of parental self-compassion have been associated with parents’ positive emotions and self-efficacy (Mancini et al., 2022). Such a physiological and emotional state is likely to increase belief in one’s abilities and expectations of success. Therefore, in this study, teachers’ self-compassion is considered a variable that can improve their sense of efficacy.

Based on the preceding review, this study aims to examine whether past maternal parenting, as perceived by teachers, affects teacher burnout, and whether teacher efficacy and self-compassion mediate that association. Specifically, three questions are analyzed: whether past parenting, as perceived by early childhood teachers, is related to self-compassion, teacher efficacy, and burnout; whether past parenting is related to burnout mediated by self-compassion and teacher efficacy; and whether teachers’ self-compassion is related to burnout mediated by teacher efficacy.

## 2 Method

### 2.1 Participants

The participants in this study were 329 early childhood teachers working at daycare centers and kindergartens in Seoul and Gyeonggi Province, South Korea, and data for the study were gathered during the period of November to December 2022. Data were collected through an online link, either sent personally or posted as a research guide on an online café for early childhood teachers. Since data collection was conducted via an online survey link, participants were unable to advance to the subsequent page in the presence of missing values. Consequently, the dataset remained free of any missing values, with untrustworthy responses being excluded from the final analysis. If they agreed to participate in the study, teachers voluntarily clicked on the link to participate. A preliminary survey was conducted with 14 early childhood teachers, who were not participants in the study, to verify any questions that were difficult to understand or had errors and to correct them. Information of teachers’ age, educational background, career length, type of working organization, class sizes, and working hours were collected.

The participant ages were as follows: 166 in their 30s (50.5%), 81 in their 40s (24.6%), 43 in their 20s (13.1%), and 39 in their 50s (24.6%). Regarding educational background, bachelor’s degrees were most common at 161 (48.9%), followed by associate degrees (99; 30.1%), and master’s degrees or higher (59; 17.9%). Regarding career length, the most common category was “more than three years and less than seven” at 127 (38.6%), followed by “more than ten years” (92; 28%), and “more than seven years and less than ten” (58; 17.6%). The largest number of teachers worked at national and corporate daycare centers at 124 (37.7%), followed by private daycare centers (86; 26.1%), national and public kindergartens (53; 16.1%), private kindergartens (33; 10%), and home daycare centers (33; 10%). Among the participants, 243 teachers (73.8%) worked at daycare centers, and 86 (26.1%) worked at kindergartens. Regarding class sizes, less than 10 children in class was the highest with 157 (47.7%), followed by classes of 10–14 children (82; 24.9%), and 25 or more children (7; 2.1%). Teachers’ working hours were asked to respond to work hours in kindergartens or daycare centers per day, and their careers were answered on a yearly basis.

### 2.2 Measure

#### 2.2.1 Maternal behavior

To measure maternal parenting, Schaefer’s Maternal Behavior Research Instrument (Schaefer and Bell, 1958), which was modified and supplemented by Jang (2020) to fit the current Korean context, was used. This scale, a classic of parenting attitudes, is a widely used measure based on parent–child interaction theories, with the advantage of measuring parenting attitudes by defining them as a specific list of behaviors. The maternal behavior scale in this study consisted of 44 questions, with 11 questions for each of the four factors: love, hostility, autonomy, and control attitude. The mean value of each 11 questions of 4 sub-factors was calculated and put into the model as the score of the corresponding factor. Responses were measured on a 5-point Likert scale, with 1 indicating “not at all” and 5 indicating “very much.” The higher the score, the higher the positive parenting attitude, or parenting that gives children affection and autonomy. In this study, the scale reliability showed a Cronbach’s ɑ coefficient of 0.85.

#### 2.2.2 Self-compassion

Neff’s (2003b) Self-Compassion Scale (SCS) was used to measure self-compassion. The scale consists of six subscales that measure the degree to which individuals display self-kindness (five questions, e.g., “I try to be loving toward myself when I’m feeling emotional pain”), against self-judgment (five questions, e.g., “I’m disapproving and judgmental about my own flaws and inadequacies”), common humanity (four questions, e.g., “I try to see my failings as part of the human condition”) versus isolation (four questions, e.g., “When I think about my inadequacies it tends to make me feel more separate and cut off from the rest of the world”), and mindfulness (four questions, e.g., “When something painful happens, I try to take a balanced view of the situation”) versus over-identification (four questions, e.g., “When I’m feeling down, I tend to obsess and fixate on everything that’s wrong”). The mean value of each questions of 3 sub-factors was calculated and put into the model as the score of the corresponding factor. In this study, the scale reliability showed a Cronbach’s ɑ coefficient of 0.81.

#### 2.2.3 Teacher efficacy

Bandura’s (2006) Teacher Self-Efficacy Scale (TSES) was used to measure teacher efficacy. The TSES consists of questions on how competent the teacher is in evaluating his/her ability to cope with various situations. In this study, 25 items were included, and five items in the “connect community” category were excluded based on the findings that the Korean context is not suitable for “connect community” due to the lack of community resources and cooperation systems (Mun and Kim, 2016). The items were measured on a 10-point Likert scale, with a higher score indicating a higher level of teacher efficacy. The mean value of each questions of 5 sub-factors was calculated and put into the model as the score of the corresponding factor. In this study, the scale reliability showed a Cronbach’s ɑ coefficient of 0.97.

#### 2.2.4 Burnout

The Maslach Burnout Inventory (MBI; Maslach et al., 1997) was used to measure burnout among early childhood teachers. It consists of 22 questions on three factors: emotional exhaustion (nine questions), feelings of cynicism (five questions), and sense of inefficiency (eight questions). The items were measured on a 5-point Likert scale, where a higher score indicates higher teacher burnout. The mean value of each questions of 3 sub-factors was calculated and put into the model as the score of the corresponding factor. In this study, the Cronbach’s ɑ for this scale was 0.94.

### 2.3 Statistical analyses

For the analysis, structural equation modeling (SEM), bootstrapping, and phantom variable modeling were conducted. Phantom variable modeling is a method of verifying the indirect effects of various parameter paths that exist when there are more than two mediated variables (Chan, 2007); it has the advantage of being able to simultaneously check the indirect effects of each mediated variable. Confirmatory factor analysis was conducted to analyze the suitability of the measurement model, and through SEM, the effect of maternal parenting as perceived by teachers on burnout was verified through self-compassion and teacher efficacy. In addition, two models were compared to confirm whether the maternal parenting affects burnout by mediating self-compassion and teacher efficacy, respectively. Otherwise, along with each mediating effect, we tried to determine whether the maternal parenting affects burnout by double mediating self-compassion and teacher efficacy. Overall, indirect effect verification was performed using a bootstrapping method, and the relationship between each compassion item was confirmed through 4 kinds of phantom variable modeling (maternal behavior-self compassion-burnout, maternal behavior-teacher efficacy-burnout, self-compassion-teacher efficacy-burnout, maternal behavior-self compassion-teacher efficacy-burnout). Frequency analysis, descriptive statistics analysis, and correlation analysis were used to examine the normality and tendency of the main variables and the reliability of the measurement. The statistical programs AMOS and SPSS 23.0 were used.

## 3 Result

### 3.1 Correlation of main variables

Table 1 presents the descriptive statistics and correlation coefficients of the main variables. The positive factors of parenting as perceived by teachers and self-compassion were positive above the median, and teacher efficacy was also high, at least 7 out of 10. However, the negative factors of parenting and burnout were lower than the median. This indicates that early childhood teachers in Korea have positive mental health characteristics. Teacher burnout is negatively related to positive parenting factors (*β* = −0.20–−0.59), self-compassion (*β* = −0.44–−0.56), and teacher efficacy (*β* = −0.17–−0.48), showing a positive association with the negative factors of parenting (*β* = 0.21–−0.43). The skewness and kurtosis of the main variables were at a level where there was no problem in analyzing the structural equation.

**Table 1:** Descriptive statistics and correlation of main variables.

	1	2	3	4	5	6	7	8	9	10	11	12	13	14	15
1	1														
2	−0.47^***^	1													
3	0.61^***^	−0.54^***^	1												
4	−0.23^***^	0.59^***^	−0.35^***^	1											
5	0.43^***^	−0.48^***^	0.50^***^	−0.33^***^	1										
6	0.34^***^	−0.37^***^	0.44^***^	−0.27^***^	0.65^***^	1									
7	0.43^***^	−0.42^***^	0.53^***^	−0.23^***^	0.75^***^	0.67^***^	1								
8	0.28^***^	−0.34^***^	0.35^***^	−0.20^***^	0.33^***^	0.30^***^	0.30^***^	1							
9	0.29^***^	−0.32^***^	0.38^***^	−0.19^***^	0.40^***^	0.29^***^	0.42^***^	0.68^***^	1						
10	0.27^***^	−0.33^***^	0.36^***^	−0.24^***^	0.37^***^	0.26^***^	0.34^***^	0.57^***^	0.79^***^	1					
11	0.26^***^	−0.37^***^	0.39^***^	−0.21^***^	0.42^***^	0.31^***^	0.38^***^	0.68^***^	0.76^***^	0.76^***^	1				
12	0.29^***^	−0.37^***^	0.41^***^	−0.22^***^	0.44^***^	0.36^***^	0.41^***^	0.61^***^	0.80^***^	0.78^***^	0.80^***^	1			
13	−0.24^***^	0.29^***^	−0.20^***^	0.21^***^	−0.52^***^	−0.44^***^	−0.49^***^	−0.17^***^	−0.30^***^	−0.27^***^	−0.25^***^	−0.29^***^	1		
14	−0.21^***^	0.43^***^	−0.36^***^	0.25^***^	−0.52^***^	−0.45^***^	−0.45^***^	−0.18^***^	−0.27^***^	−0.26^***^	−0.31^***^	−0.35^***^	0.57^***^	1	
15	−0.44^***^	0.41^***^	−0.59^***^	0.21^***^	−0.55^***^	−0.44^***^	−0.56^***^	−0.35^***^	−0.46^***^	−0.45^***^	−0.43^***^	−0.48^***^	0.28^***^	0.49^***^	1
M	3.26	2.48	3.42	2.83	3.33	3.30	3.29	7.39	7.33	7.71	7.72	7.88	2.65	1.91	2.44
SD	0.74	0.68	0.64	0.66	0.56	0.52	0.56	1.62	1.19	1.20	1.33	1.19	0.84	0.75	0.63
Skewness	−0.08	−0.10	0.11	−0.13	0.21	0.27	0.33	−0.61	−0.43	−0.43	−0.41	−0.47	0.01	0.55	0.24
Kurtosis	0.20	−0.49	−0.28	−0.15	0.26	0.60	0.37	0.39	0.30	−0.01	0.14	0.01	−0.38	−0.53	0.72

^***^p < 0.001 All correlation coefficients are significant at 0.001 level.

1. Maternal behavior_love. 2. Hostility. 3. Autonomy. 4. Control. 5. Self compassion_self-kindness. 6. Universal humanity 7. Mindfulness. 8. Teacher efficacy_decision making. 9. Instructional efficacy. 10. Disciplinary. 11. Family connection. 12. Learning climate. 13. Burnout_emotional exhaustion. 14. Feeling cynicism. 15. Sense of inefficiency.

### 3.2 Measurement model

**Figure 1:**
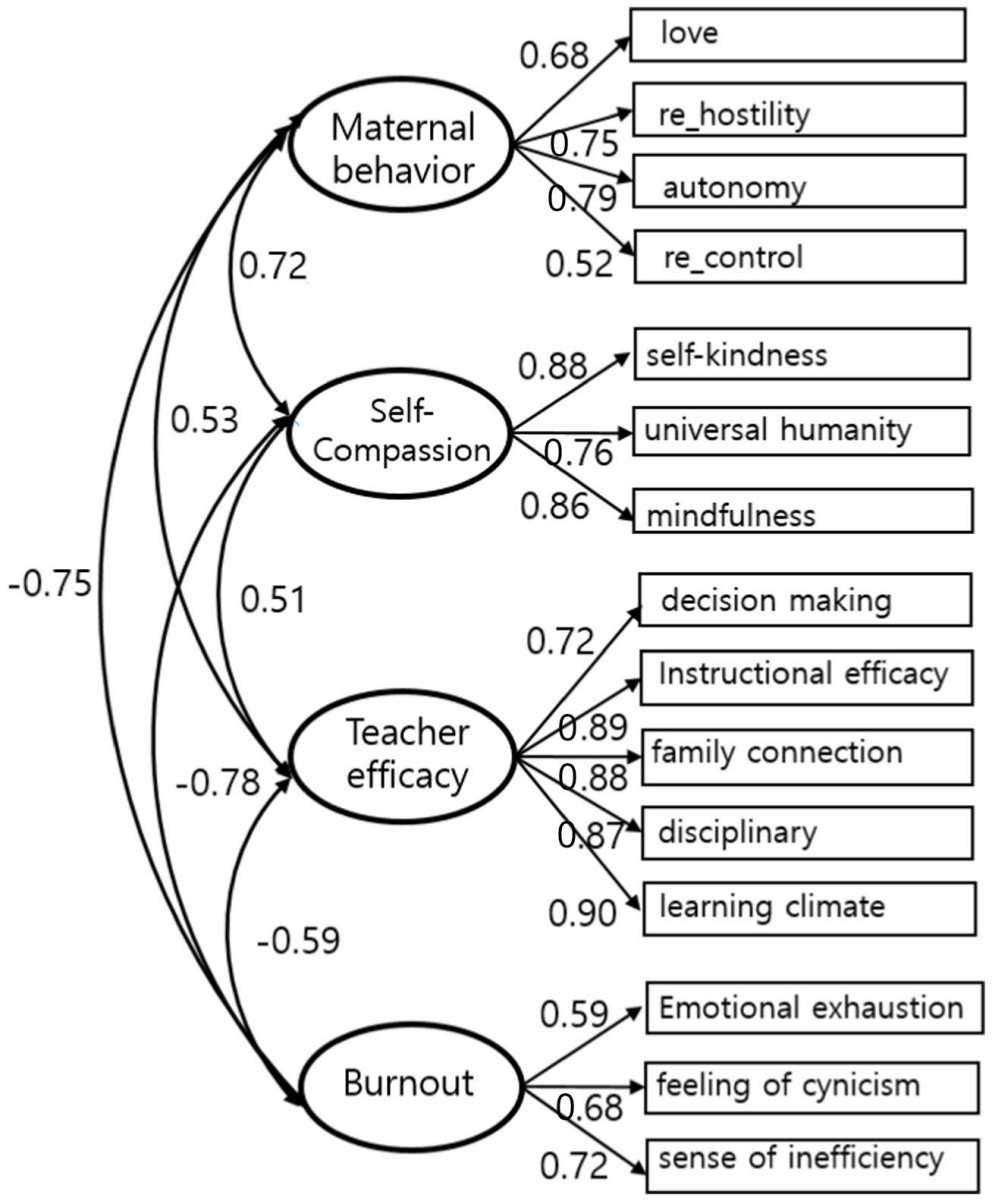
Measurement model.

In SEM, before examining the influence of the variables, it is necessary to evaluate whether the concepts included in the research model are properly estimated. Therefore, it was confirmed through SEM analysis that the indicators of maternal parenting, self-compassion, teacher efficacy, and burnout theoretically reflect the concept of the potential variables. The standardization coefficient values for each path of the latent and measurement variables ranged from 0.52 to 0.90, which was judged as appropriate. In addition, all values were statistically significant at the 0.001 level, and it was confirmed that all measurement variables explained the latent variables well. The *χ*^2^ value of the measurement model was 348.72 (*df* = 84, *p* = 0.000), which is significant at the 0.001 level, and the model fit was also acceptable in Figure 1 (NFI = 0.90, CFI = 0.92, TLI = 0.90, RMSEA = 0.09).

### 3.3 Structure model comparison

This study seeks to determine whether maternal behavior is directly related to teacher efficacy, teacher burnout, and self-compassion, or whether self-compassion has a mediating effect on burnout through teacher efficacy. Through a model comparison, the final model was selected among the two models. In Model 1, maternal behavior affected self-compassion, teacher efficacy, and burnout, whereas self-compassion and teacher efficacy have a mediating effect on burnout. In Model 2, the double mediating effect of self-compassion was added to Model 1, which relates to exhaustion through teacher efficacy.

**Figure 2:**
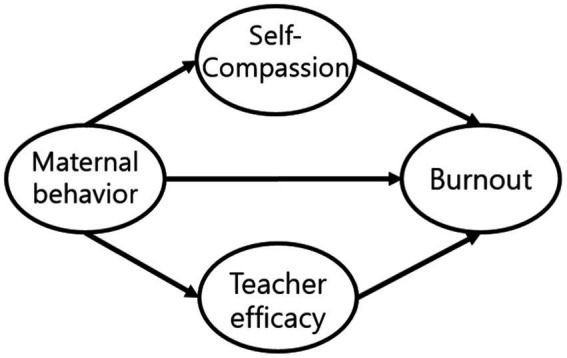
Model 1.

The comparison results of the two models are presented in Table 2. The degrees of freedom and *χ*^2^ values of each model, and the fit of each model were compared based on the normed fit Index (NFI), Tucker-Lewis index (TLI), comparative fit index (CFI), and root mean square error of approximation (RMSEA). The NFI, TLI, and CFI are relative fit indices that show how well the theoretical model explains the data compared with the base model. RMSEA is an absolute fit index that evaluates how well the theoretical model fits the data. If the value of χ^2^ that the added path can reduce is significant at 1 degree of freedom (*p* = 0.05, *χ*^2^ value = 3.838), the path is worth considering on a statistical basis. In general, RMSEA shows a reasonable fit below 0.08, and an NFI, TLI, and CFI above 0.9 are evaluated as a good fit (Browne and Cudeck, 1993). Each time a path was added in Model 1, the *χ*^2^ value significantly decreased, and the model fit index also tended to increase. The χ^2^ value of Model 1 was 463.08, and the model fit was 0.9 for NFI, TLI, and CFI, while the RMSEA was 0.09. The *χ*^2^ value was significantly reduced in Model 2 (Δ*χ*^2^ = 9.41), and the model fit was slightly better overall than Model 1. Consequently, Model 2 was selected as the final model for this study in Figure 2.

**Table 2:** Model comparison.

	df	*χ*^2^	NFI	CFI	TLI	RMSEA
Model 1	114	463.08	0.89	0.90	0.91	0.09
Model 2	113	453.67	0.89	0.91	0.92	0.08
△	1	9.41	−	0.01	0.01	0.01

**Figure 3:**
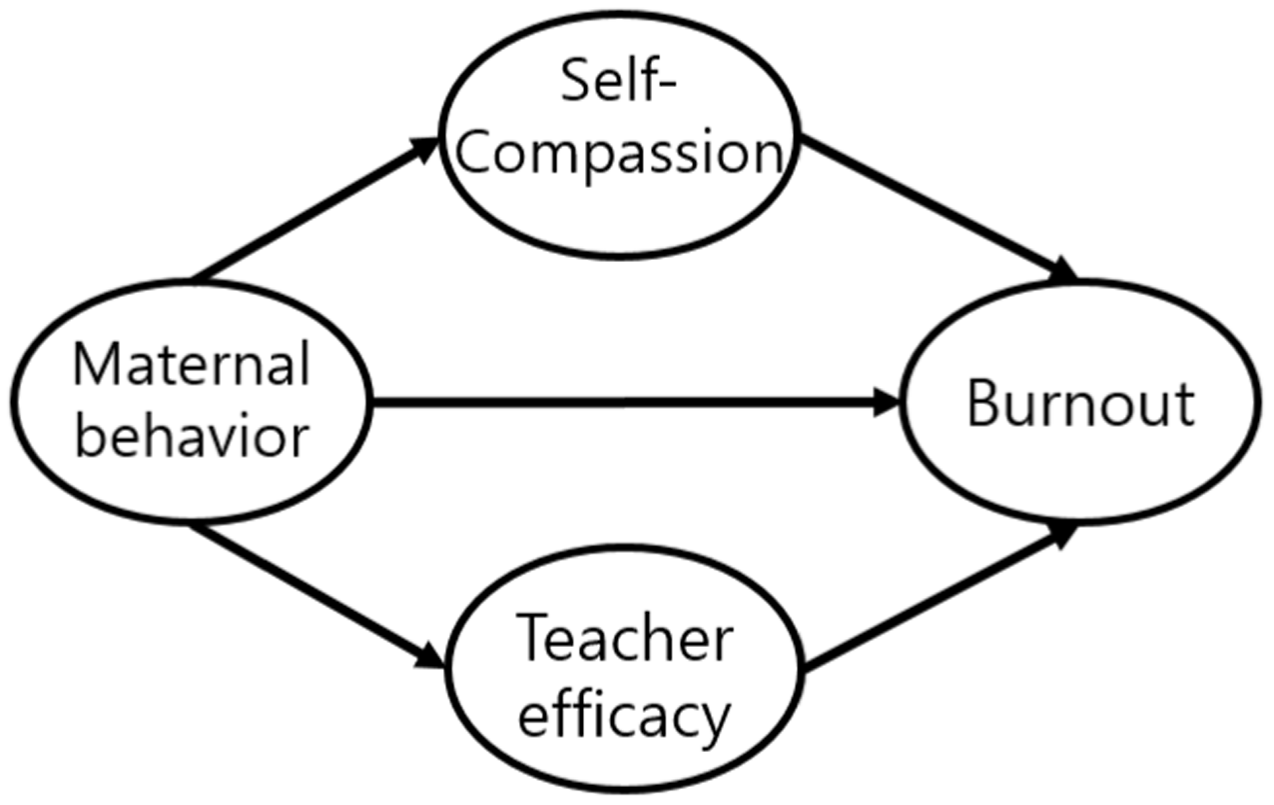
Model 2.

**Figure 4:**
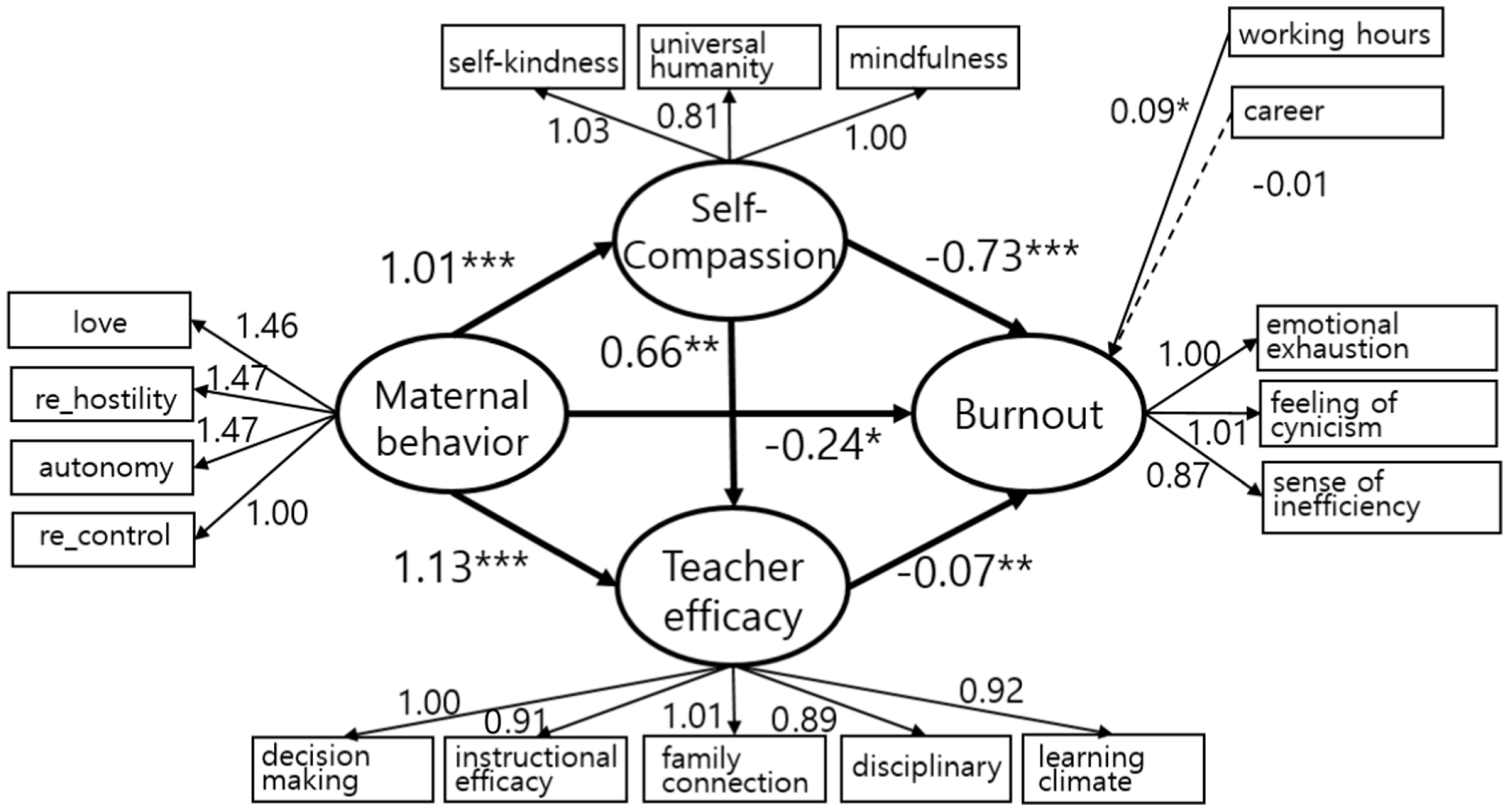
Final Model. Unstandardized path coefficients are reported. The loading of all observed variables was significant at *p* < 0.001 level. *p < 0.05, **p < 0.01, ***p < 0.001.

The final model used in this study is shown in Figure 3 and Table 3. According to previous studies (Gavish and Friedman, 2010; Baeriswyl et al., 2021), working hours and career length are known to affect teacher burnout and were used as control variables. Maternal behavior was found to be directly related to self-compassion (*B* = 1.01, *p* < 0.001), teacher efficacy (*B* = 1.13, *p* < 0.001), and burnout (*B* = -0.24, *p* < 0.05). Maternal behavior and burnout were double mediated by self-compassion and teacher efficacy. Moreover, working hours were associated with burnout while career length was not in Figure 4.

**Table 3:** Direct and indirect effects between latent variables.

Path	Direct effect (β)	Indirect effect (β)	Total effect (β)
Maternal behavior ⇒ Self-compassion	72^***^		0.72^***^
maternal behavior ⇒ Teacher efficacy	0.33^***^	0.20^*^	0.53^***^
teacher efficacy ⇒ Burnout	−0.15^**^		−0.15^*^
self-compassion ⇒ Burnout	−0.69^***^	−0.04^*^	−0.73^***^
self-compassion ⇒ Teacher efficacy	0.27^**^		0.27^**^
maternal behavior ⇒ Burnout	−0.16^*^	−0.57^***^	−0.73^***^

### 3.4 Mediator analyses

The size and significance of the mediating effect were tested using the bootstrapping method in Table 4. Bias-corrected bootstrapping with 2,000 repetitions was performed on the estimate of the mediating model to calculate a 95% confidence interval, and the mediating effect was determined to be significant if there was no zero between the lower and upper limits of the confidence interval.

**Table 4:** Verification of each mediated effect by phantom model.

Path	B	S. E.	Bias-corrected	
			Lower	Upper
Maternal behavior ⇒ Self-compassion ⇒ Burnout	−0.74^**^	0.18	−1.19	−0.46
Maternal behavior ⇒ Teacher efficacy ⇒ Burnout	−0.07^*^	0.04	−0.19	−0.02
Maternal behavior ⇒ Self-compassion ⇒ Teacher efficacy	0.66^**^	0.23	0.27	1.22
Self-compassion ⇒ Teacher efficacy ⇒ Burnout	−0.04^*^	0.02	−0.11	−0.01
Maternal behavior ⇒ Self-compassion ⇒ Teacher efficacy ⇒ Burnout	−0.04^*^	0.02	−0.10	−0.01

B, unstandardized coefficient; S.E., standard error.

Finally, nine phantom variables were set to verify the significance of each mediating effect and bootstrapping was performed with a 95% confidence interval. The phantom variable is a hypothetical variable that does not affect the model fit or parameter values. The results indicated that in the relationship between teacher-perceived parenting and burnout, both the mediation and double mediation of self-compassion and teacher efficacy were statistically significant. Thus, maternal parenting was confirmed to be related to burnout through the sequential mediation of self-compassion and teacher efficacy.

## 4 Discussion

This study was conducted to confirm the importance of maternal parenting and self-compassion as perceived by early childhood teachers in a context where the significance of early childhood teachers is increasing. The main results are as follows.

First, positive parenting, self-compassion, and teacher efficacy were positively correlated with one another and negatively correlated with burnout. This is similar to previous studies that helicopter parenting that violates children’s autonomy had a negative effect on the burnout of adult children (Love et al., 2020). indicating that negative maternal parenting is related to higher levels of burnout in children, and that positive parenting and an autonomous and supportive climate prevent burnout in children (Álvarez et al., 2019). Maternal parenting based on warm affection that supports a child’s autonomy exposes the child to an appropriate level of risk and adversity from an early age, and the child develops a sense of efficacy through the experience of overcoming adversity. Teachers who perceive themselves as efficient and successful experienced low levels of burnout (Aloe et al., 2014; Zhu et al., 2018). This study also supports the findings of previous studies (Zhao et al., 2021; Dakers and Guse, 2022) that warm and responsive parenting is related to self-compassion, where teachers treat themselves kindly.

Second, the model comparison shows that positive parenting is negatively related to burnout, and positive parenting is negatively related to burnout through the mediation of self-compassion and teacher efficacy. Few if any previous studies focus on teacher efficacy, self-esteem, and burnout in a similar context. Teachers’ self-compassion mediated parenting and teacher efficacy, and as a result, burnout was double-mediated. In other words, self-compassion and teacher efficacy partially mediated the effect of maternal parenting on burnout. Positive perceptions of maternal parenting lowered burnout by increasing teachers’ self-compassion and efficacy, and positive maternal parenting lowered burnout by increasing self-compassion and, consequently, increasing teacher efficacy.

Comparing the standardized coefficient of the direct effect in the final model of this study, it was found that teachers’ self-compassion was most closely related to teacher burnout. The coefficient of teacher efficacy was very small compared to that of self-compassion. These results suggest that early childhood teachers’ self-compassion is an important variable in relation to burnout. Self-compassion is likely to affect comfortable emotions and can play a role in alleviating emotional exhaustion, which is a sub-factor of burnout. According to previous studies on self-compassion, higher acceptance of oneself also increases the acceptance of others, improving the quality of interpersonal relationships (Neff, 2003b). Only when you accept and recognize that you are not perfect can others also recognize that you are not perfect (Zhang et al., 2020), and self-compassion can eventually be extended to compassion toward others. People with a high level of self-compassion have smooth family and interpersonal relationships (Amani and Khosroshahi, 2020), which are likely to alleviate the feelings of cynicism, a sub-factor of burnout. Self-compassion can also alleviate the sense of inefficiency, which is a subfactor of burnout. This is because individuals can strive toward achievement by acknowledging their imperfections and viewing the experience of failure or frustration as something that anyone can experience in life.

The self-compassion of early childhood teachers also had a positive relationship with teacher efficacy, which supports the argument that individuals expect success when they are emotionally stable, and that their efficacy increases when negative emotional tendencies are low (Bandura, 1991). Moreover, increased teacher efficacy due to self-compassion plays a role in lowering burnout. This supports the findings of previous studies (Akpan and Saunders, 2017), which suggest that teachers should treat themselves kindly, and that this process may affect teacher efficacy.

The following suggestions derive from this study’s findings. First, teachers must pay attention to self-compassion. A considerable number of studies on early childhood teachers are focused on variables measuring the success of their teacher roles, rather than focusing on the psychological or emotional state of teachers. Such studies are interested in how teachers perform their teaching role, teaching efficacy, expertise, teaching methods, and dynamics within kindergartens or daycare centers. However, self-compassion is a malleable trait that can be cultivated and strengthened through training and practice (Irons, 2013; Neff and Germer, 2013; Halamová et al., 2021). Thus, self-compassion is an important factor in promoting teachers’ psychological health and is an important conceptual component of psychotherapy (MacBeth and Gumley, 2012). Considering that early childhood teachers are also people before becoming teachers, it is necessary to pay more attention to their psychology and emotions, as well as variables related to their role as teachers. At a time when the stress of early childhood teachers is increasing daily, self-compassion is a concept worthy of attention.

Only someone who acknowledges his or her imperfections and knows how to take good care of themselves can acknowledge and care for others. As acceptance of oneself increases, acceptance of others also increases (Neff, 2003a), and warm acceptance is the most necessary virtue for early childhood teachers. Infants and children are less developed than adults; therefore, their behavior and expression of emotions may be immature and clumsy. Compassion, which endlessly loves and pities infants and children, is an essential element for early child teachers. Only good people who love themselves and are kind to themselves can be good teachers, and this premise is more absolute for teachers dealing with small children.

Second, specific programs or support are required to promote early childhood teachers’ self-compassion. Such programs may include content that can be executed individually after a certain training period, and are even effective as short-term online programs (Halamová et al., 2021). Self-compassion training, such as meditation practice, repeating or writing self-compassion phrases, creating new self-compassion phrases, expressive writing, and self-compassion writing about one’s experiences, are also helpful. Self-compassion training can be seen as a positive psychological training that fosters “warm me” in the mind. Finally, self-compassion focuses on the phrase compassion wishing for happiness and peace, stating it repeatedly, and in the process, other desires and thoughts unrelated to compassion must be put down. Previous studies have shown that six to eight sessions of self-compassion programs, which are not lengthy, have significantly helped participants practice self-compassion even after the program’s end and have played a positive role in continuing their charitable attitudes in the future (Neff and Germer, 2013). In addition, a program to embody “mindfulness” can also be useful. This means that when you encounter painful thoughts and feelings, you do not force them down or exaggerate them but take a step away and observe them critically (Neff, 2003b). This is meant to not avoid or hide suffering, but to carefully observe one’s experience in the “here and now” and fully experience the pain (Neff, 2003a; Germer, 2009). Considering that many educational programs for early childhood teachers are not of practical help after education and are quickly forgotten, and that teachers’ self-compassion is more closely related to burnout than teacher efficacy, the need for self-compassion programs for early childhood teachers is even more urgent.

One limitation of this study is that data was collected through a self-report questionnaire from early childhood teachers and answered by recalling their maternal parenting. Future studies should collect data through observation or experiments. Given potential variations in perceptions and treatment of early childhood teachers, it is imperative to conduct research across diverse cultural and national contexts. While the model employed in this study was deemed acceptable, it exhibited insufficient model fit. Given that research on self-compassion among early childhood teachers, which profoundly influences the well-being of children, is still in its nascent stage, it becomes imperative to carefully choose scale items that are culturally relevant and to develop a robust theoretical model. Despite this limitation, this study is meaningful in suggesting the importance of self-compassion for early childhood teachers and suggests that self-compassion is more strongly related to burnout than teacher efficacy. Therefore, when considering teacher burnout-related interventions, focusing on psychological and internal variables, such as self-compassion, rather than teacher-related variables, such as teacher efficacy, is recommended. Only a compassionate person can become a compassionate and effective early childhood teacher.

## Data availability statement

The original contributions presented in the study are included in the article/supplementary material, further inquiries can be directed to the corresponding author.

## Ethics statement

The studies involving humans were approved by Gachon University 1044396-202212-HR-222-01. The studies were conducted in accordance with the local legislation and institutional requirements. The participants provided their written informed consent to participate in this study.

## Author contributions

YJ designed and executed the study, assisted with the data analyses, and wrote the manuscript. Y-JH collaborated in designing the study, executing the study, and writing and editing the final manuscript. All authors contributed to the article and approved the submitted version.
